# Meta-Analysis: Characteristics of Retinal Vasculature in Obstructive Sleep Apnea Syndrome Humans

**DOI:** 10.1155/2024/4600428

**Published:** 2024-07-16

**Authors:** Kaibao Ji, Yang Yang, Qinglin Zhang, Yiqiao Xing, Wei Wan

**Affiliations:** ^1^ Department of Ophthalmology Renmin Hospital of Wuhan University, Wuhan, Hubei, China; ^2^ Department of Ophthalmology Huangshi Central Hospital Affiliated Hospital of Hubei Polytechnic University Edong Healthcare Group, Huangshi, China

## Abstract

**Background:**

The objective of this study is to determine optic nerve head vascular changes in patients with obstructive sleep apnea-hypopnea syndrome (OSAS) by utilizing an optical coherence tomography angiography (OCTA) device.

**Methods:**

A detailed studies search was screened in the PubMed, Embase, the Cochrane Library, and Web of Science databases from inception to August 2023. We reviewed and examined optic nerve head vascular density in eyes with OSAS and controls. The mean difference and 95% confidence interval were calculated to evaluate continuous outcomes. Review Manager version 5.4.1 was applied for analysing pooled data.

**Results:**

Six eligible studies were included in our meta-analysis. The radial peripapillary capillary (RPC) whole enface vessel density (VD) measured by OCTA in the mild-to-moderate and severe OSAS groups was significantly lower compared to the controls (MD = −0.96, *P* = 0.03; MD = −1.42, *P* = 0.001, respectively). For RPC peripapillary VD, eyes in mild-to-moderate OSAS showed a trending decrease compared to the controls (MD = −1.71, *P* = 0.05), and there was a remarkable difference between eyes with severe OSAS and the controls (MD = −3.08, *P* = 0.004). In addition, the RPC inside disc VD was decreased in severe OSAS eyes than in the controls (MD = −0.07, *P* = 0.94).

**Conclusions:**

Our results revealed that peripapillary vascular density was attenuated in patients with OSAS. Moreover, on the basis of these findings, we suggest that optic nerve head vascular density measured by OCTA may be used as a potential tool to diagnose and monitor the severity of patients with OSAS.

## 1. Introduction

Obstructive sleep apnea-hypopnea syndrome (OSAS) is a respiratory sleep-related illness characterized by repetitive partial or complete upper airway obstruction during sleep, resulting in recurrent hypoxia and fragmented sleep. Accumulating epidemiologic studies have demonstrated that the number of people affected worldwide with OSAS is almost 1 billion, and the incidence of OSAS is estimated to be 24% in men and 9% in women among the population aged 30–60 years [1–3]. Multiple risk factors that increase the development of OSAS include obesity, male sex, age, ethnicity, and smoking [4, 5]. The underlying pathogenesis of OSAS is incompletely elucidated. Evidence recognized that anatomical factors, neuromuscular factors, and pharyngeal collapsibility and instability of central motor output and arousal thresholds are probably involved in the pathophysiology of OSAS [6, 7].

Accumulating studies have documented that OSAS was correlated with several neurological disorders, including multiple sclerosis, Alzheimer's disease, and Parkinson's disease [8–10]. In addition, studies have reported that OSAS has been associated with some ocular conditions such as nonarteritic anterior ischemic optic neuropathy, retinal vein occlusion, central serous chorioretinopathy, and glaucomatous optic neuropathy [11, 12]. For people with OSAS, recurrent apnea can lead to transient hypoxemia and increase vessel resistance, which subsequently contributes to optic nerve head (ONH) hypoperfusion and poor blood flow [13]. Recently, optical coherence tomography angiography (OCTA) is a noninvasive imaging facility which can quantify the analysis of blood flow changes within the retinal microvascular network [14]. Furthermore, several current studies conducted by OCTA have revealed that patients with OSAS possessed decreased vessel density in both ONH and parafoveal area [15, 16], whereas others did not detect reduced ONH and macular vascular density in eyes with OSAS [17]. However, to date, there has been no meta-analysis comprehensively examining optic disc head vascular density in the patients with OSAS.

Therefore, to address the abovementioned mixed issue, we attempted to conduct a meta-analysis to evaluate alterations in retinal microvasculature in patients with OSAS and offer abundant evidence for clinicians to understand and assess patients with OSAS.

## 2. Methods

### 2.1. Search Strategy

The current meta-analysis was carried out in accordance with the Preferred Reporting Items for Systematic Reviews and Meta-Analyses (PRISMA) guideline [18]. As prior published studies were analysed, ethical approval was not needed. Two independent authors (Kaibao Ji and Yang Yang) systematically searched the articles from inception to August 2023 by using PubMed, Embase, the Cochrane Library, and Web of Science electronic databases. The following combination of search terms was employed to each database: ((((OCTA) OR (OCT angiography)) OR (optical coherence tomography angiography)) OR (optical coherence tomographic angiography)) AND ((((obstructive sleep apnea syndrome) OR (obstructive sleep apnea)) OR (obstructive sleep hypopnea)) OR (OSAS)). Language restricted in English was considered eligible. Any disparities between the two investigators were completed by discussing with each other or consulting with the third author.

### 2.2. Study Selection

Suitable studies had to fulfill the following criteria: (1) original case-control studies; (2) patients with OSAS in comparison with healthy controls; (3) OCTA data for calculation should be shown as mean ± standard deviation (SD); and (4) observed metrics including radial peripapillary capillary (RPC) whole enface vessel density, RPC peripapillary VD, and RPC inside disc VD.

Studies were excluded if they met the following characteristics: (1) case reports, conference abstracts or posters, reviews or meta-analyses, nonhuman studies, and comments; (2) non-English written studies; (3) the article results were not suitable for calculating; (4) duplicated studies; and (5) the measured parameters did not meet the inclusion characteristics.

### 2.3. Data Collection and Quality Assessment

Two independent reviewers (Kaibao Ji and Yang Yang) collected the data from the eligible studies, and any discrepancies in the data extraction process were resolved by discussion. The following data were retrieved from the included articles: first author and publication date, study type, numbers of enrolled eyes, mean age, sex, OCTA software, measurement outcomes, and study quality analysis. The methodological quality of the included articles was judged in accordance with the Newcastle–Ottawa Scale (NOS) for the quality of case-control studies, with a score range of 0–9 stars, and studies with ≥5 stars were indicated as having adequate quality [19].

### 2.4. Statistical Analysis

RevMan Software version 5.4.1 (Cochrane Collaboration, Oxford, UK) was adopted to perform statistical analysis. OCTA metrics for calculation should be reported in the mean ± standard deviation (SD) format. The pooled difference in vessel density between OSAS and control groups was calculated by mean difference (MD) with a confidence interval (CI) of 95%. The sample mean and standard deviation were estimated according to previous studies reported [20, 21]. Statistical heterogeneity across the studies was conducted by the chi-square test and *I*^2^ statistic. *I*^2^ value >50% (or *P* < 0.01) indicated significant heterogeneity, and a random effect model was adopted for meta-analysis. Otherwise, a fixed-effect model was utilized. A funnel plot was adopted to evaluate potential publication bias. A *P* value <0.05 indicated a significant difference.

## 3. Results

### 3.1. Literature Search

**Figure 1:**
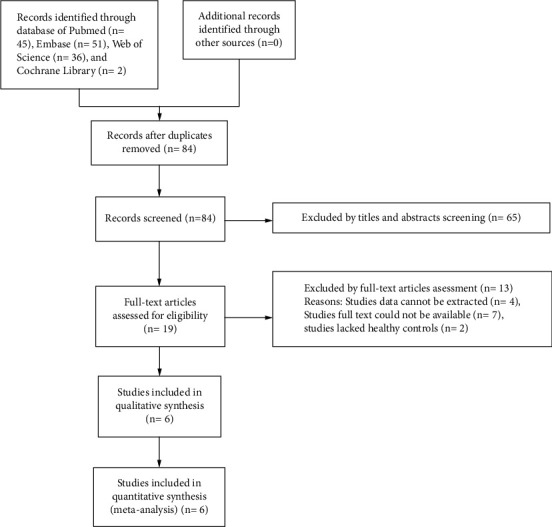
Study selection process of the meta-analysis.

The literature selection process is shown in Figure 1. A total of 134 records were initially identified from the electronic databases (Embase: 51, PubMed: 45, Web of Science: 36, and Cochrane Library: 2), of which 50 studies were excluded due to duplicates. The remaining records were screened by the titles and abstracts, which led to 65 irrelevant records being removed. In addition, the residual 19 records were thoroughly assessed for the full text. In this phase, seven studies were omitted because they were unavailable full-text articles. Two studies were excluded due to the lack of control groups. Four studies were removed since the data cannot be extracted. Finally, the remaining 6 eligible articles [16, 17, 22–25], containing 479 eyes (333 in the OSAS group and 146 in the control group) were included in this meta-analysis.

The main characteristics of the eligible studies are shown in Table 1, and the quality of these articles is presented in Table 2.

### 3.2. Meta-Analysis

**Figure 2:**
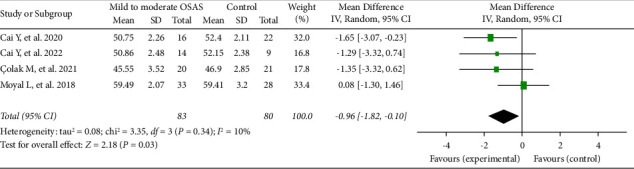
Forest plot for RPC whole enface vessel density between eyes with mild-to-moderate OSAS and controls.

**Figure 3:**
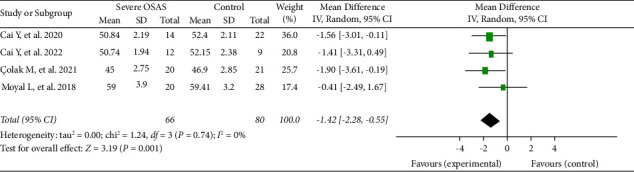
Forest plot for RPC whole enface vessel density between severe OSAS and control groups.

Four studies, containing 163 eyes (83 in the mild-to-moderate OSAS group and 80 in the control group), reported on the RPC whole enface vascular density. The pooled results revealed that the RPC whole enface VD was significantly decreased in the mild-to-moderate OSAS group than in that of the control group (MD = −0.96, 95% CI: −1.82 to −0.10, *P*=0.03), as described in Figure 2. We also analysed the difference between the severe OSAS group and the controls. Four eligible articles, including 146 eyes (66 in the severe OSAS group and 80 in the control group), showed that the pooled mean difference for RPC whole enface VD between the OSAS and control groups was −1.42 (95% CI: −2.28 to −0.55, *P*=0.001, Figure 3), with no heterogeneity across studies (chi^2^ = 1.24, *P*=0.74, *I*^2^ = 0%, Figure 3), indicating that the RPC whole enface VD was reduced in the patients with severe OSAS.

**Figure 4:**
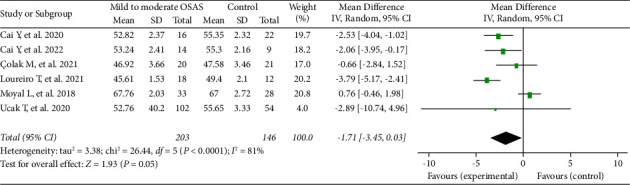
Forest plot analysis of RPC peripapillary vessel density in mild-to-moderate OSAS patients and controls.

**Figure 5:**
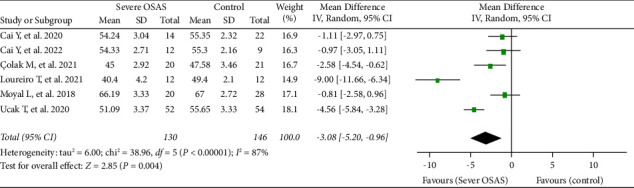
Forest plot for RPC peripapillary vessel density between eyes with severe OSAS and controls.

In addition, six studies, involving 349 eyes (203 in the mild-to-moderate OSAS group and 146 in the control group), evaluated the RPC peripapillary VD between the OSAS and healthy control groups. The summary MD was nearly significant between the two groups (MD = −1.71, 95% CI: −3.45 to 0.03, *P*=0.05, Figure 4), with substantial heterogeneity among studies (chi^2^ = 26.44, *P* < 0.0001, *I*^2^ = 81%, Figure 4). Besides, six other studies reported the RPC peripapillary VD between the severe OSAS group and the healthy control group. The pooled results indicated that the RPC peripapillary VD was significantly lower in the eyes with severe OSAS than that of the controls (MD = −3.08, 95% CI: −5.20 to −0.96, *P*=0.004, Figure 5), but a high heterogeneity existed across the studies for this result (chi^2^ = 38.96, *P* < 0.00001, *I*^2^ = 87%, Figure 5).

**Figure 6:**
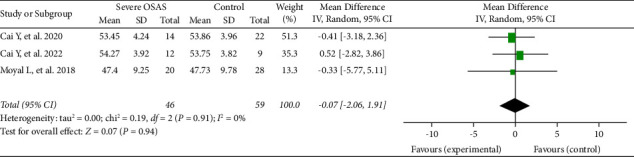
Forest plot analysis of RPC inside disc vessel density between severe OSAS and control groups.

Furthermore, three studies analysed the RPC inside disc VD in 105 eyes (46 eyes in the severe OSAS group and 59 eyes in the control group). Among these studies, the RPC inside disc VD was lower, not significant, in OSAS eyes (MD = −0.07, 95% CI: −2.06 to 1.91, *P*=0.94, Figure 6) and showed a low heterogeneity (chi^2^ = 0.19, *P*=0.91, *I*^2^ = 0%, Figure 6).

### 3.3. Publication Bias

**Figure 7:**
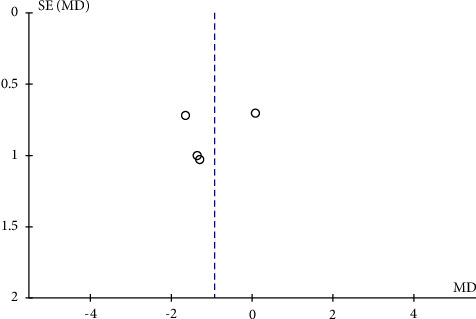
A funnel plot of RPC whole enface VD between eyes with mild-to-moderate OSAS and controls indicating no substantial publication bias. SE: standard error; MD: mean difference.

**Figure 8:**
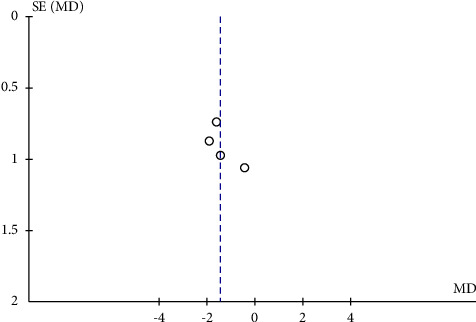
A funnel plot of RPC whole enface VD between severe OSAS and control groups showing no significant publication bias. SE: standard error; MD: mean difference.

**Figure 9:**
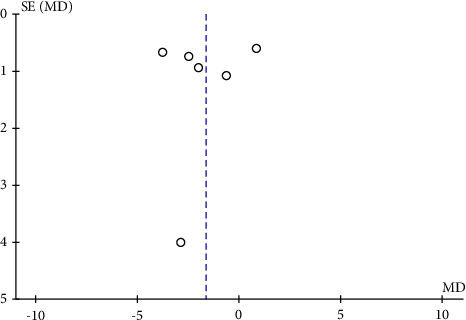
A funnel plot of RPC peripapillary vessel density in mild-to-moderate OSAS patients and controls revealing no remarkable publication bias. SE: standard error; MD: mean difference.

**Figure 10:**
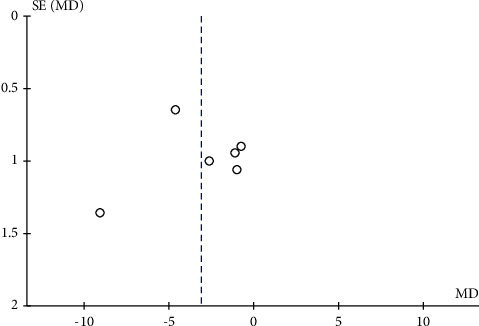
A funnel plot of RPC peripapillary vessel density in severe OSAS eyes and controls demonstrating no notable publication bias. SE: standard error; MD: mean difference.

**Figure 11:**
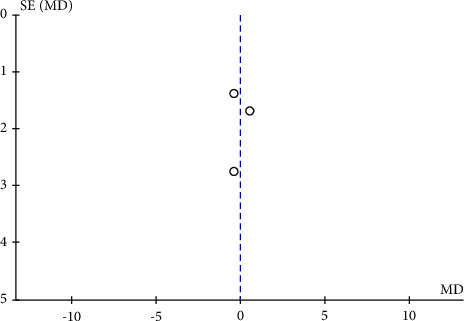
A funnel plot of RPC inside disc VD between severe OSAS and control groups showing no significant publication bias. SE: standard error; MD: mean difference.

The potential publication bias of RPC whole enface VD, RPC peripapillary VD, and RPC inside disc VD across the studies was assessed by the funnel plots. The results showed that no obvious evidence of publication bias was found in the total analyses of vascular density (Figures 7, 8, 9, 10, and 11).

## 4. Discussion

To date, this is the first meta-analysis to calculate changes in retinal microvasculature density measured by OCTA in patients with OSAS and healthy controls. Six eligible articles containing 479 eyes (333 in the OSAS group and 146 in the control group) were analysed in this meta-analysis. In the review, we analysed the mean RPC whole enface, RPC peripapillary, and RPC inside disc vessel densities of study participants. We figured out that the pooled mean for the RPC whole enface, RPC peripapillary, and RPC inside disc vascular densities, were decreased in eyes with OSAS compared to the control group. The funnel plots revealed no obvious evidence of publication bias.

It was reported that OSAS has been associated with certain ocular disorders such as nonarteritic anterior ischemic optic neuropathy, retinal vein occlusion, central serous chorioretinopathy, and glaucomatous optic neuropathy [11, 12]. OCTA is a nonaninvasive imaging technology which can quantify the analysis of blood flow alterations within retinal microvascular capillary [26, 27]. Several previous studies conducted by OCTA revealed that the reduction of vessel density in the optic nerve head was found in patients with OSAS [15, 16]. The pooled RPC whole enface VD was significantly decreased in the mild-to-moderate OSAS patients than that of controls (*P*=0.03, Figure 2). We also concluded that the pooled RPC whole enface VD was substantially lower in the severe OSAS group compared with the control group (*P*=0.001, Figure 3). Our current data strongly consolidated the results of prior studies [22, 24]. Besides, no obvious heterogeneity was found in our meta-analysis (chi^2^ = 3.35, *I*^2^ = 10%; chi^2^ = 1.24, *I*^2^ = 0%, respectively), as described in Figures 2 and 3.

In terms of RPC peripapillary VD meta-analysis, the *I*^2^ measurement for the mild-to-moderate OSAS group was notably decreased from 81% to 36% (chi^2^ = 6.20, *P* = 0.18) after reference 17 was ruled out. One potential factor was the relatively high mean VD reported in this study. Regarding the analysis of RPC peripapillary VD in the severe OSAS group and controls, we found that remarkable heterogeneity existed even after excluding the study by Moyal et al. [17]. Relatively small sample size, ethnic difference, and comparatively lower quality of the evidence may have elicited the high heterogeneity in this analysis.

OSAS is characterized by recurrent obstruction of the upper airway and a decrease in oxygen saturation during sleep [13]. The reduction of vascular density of ONH in OSAS can be explained by repeated apnea leading to transient hypoxemia [13], decrease in the arterial oxygen partial pressure and elevation of arterial carbon dioxide partial pressure [28], temporarily increased vascular resistance, and hypoperfusion in ONH blood flow [29]. In addition, chronic intermittent hypoxemia contributed to the disequilibrium of arginase-endothelial nitric oxide synthase and enhanced expression of endothelin-1, leading to vascular remodeling and ischemia [30, 31].

The present meta-analysis may have several noteworthy limitations. First, the relatively small sample sizes included in the study and the comparatively lower quality of the evidence. Second, caution should be taken in interpreting the pooled results due to the high heterogeneity existing among the studies. Third, we could not conduct a meta-regression to completely determine the source of heterogeneity due to a lack of sufficient data. Finally, although we did not register our study's protocol in the PROSPERO database, no corresponding protocols were identified in the database. To confirm our findings, prospective longitudinal studies with adequate sample sizes are required to assess ONH vasculature features in patients with OSAS in the future.

In conclusion, our current results demonstrated that peripapillary vascular density was attenuated in patients with OSAS. Moreover, on the basis of these findings and the noninvasive nature of OCTA imaging facility, we suggest that the measurement of optic nerve head vascular density by OCTA may presumably serve as a potential biomarker to objectively monitor and diagnose OSAS in the future.

## Figures and Tables

**Table 1:** The baseline characteristics of eligible articles.

Article	Place	Age (years)	Design	Eyes	OCTA manufacturer	Scan area (mm^2^)	Primary outcomes
Ucak and Unver 2020 [16]	Turkey	52.17 ± 11.36/55.91 ± 8.96/55.42 ± 9.44 52.89 ± 11.60	Prospective study	Mild/moderate/severe OSAS: 54/48/52 Control: 54	Nidec	2.4 × 4 in ONH	RPC peripapillary VD
Moyal et al. 2018 [17]	France	50.4 ± 11.9/53.2 ± 12.7/54.4 ± 12.1 48.9 ± 15.5	Retrospective observational study	Mild/moderate/severe OSAS: 16/17/20 Control: 28	Optovue	3 × 3 in ONH	RPC whole enface VD, RPC peripapillary VD, and RPC inside disc VD
Cai et al. 2020 [22]	China	42.38 ± 9.41/45.43 ± 11 43.73 ± 11.23	Observational study	Mild-to-moderate OSAS/severe OSAS: 16/14 Control: 22	Optovue	4.5 × 4.5 in ONH	RPC whole enface VD, RPC peripapillary VD, and RPC inside disc VD
Çolak et al. 2021 [23]	Turkey	49.8 ± 8.9/51.9 ± 9.54 45 ± 5.7	Prospective case-control study	Mild-to-moderate OSAS/severe OSAS: 20/20 Control: 21	Optovue	NA	RPC whole enface VD and RPC peripapillary VD
Cai et al. 2022 [24]	China	37.29 ± 8.00/43.33 ± 9.06 41.78 ± 12.13	Prospective observational study	Mild-to-moderate OSAS/severe OSAS: 14/12 Control: 9	Optovue	4.5 × 4.5 in ONH	RPC whole enface VD, RPC peripapillary VD, and RPC inside disc VD
Loureiro et al. 2021 [25]	Portugal	70.5 ± 9.9/68 ± 7.6/65.9 ± 3.9 66.4 ± 5.2	Retrospective study	Mild/moderate/severe OSAS: 8/10/12 Control: 12	Zeiss	3 × 3 in ONH	RPC peripapillary VD

**Table 2:** Study quality evaluated using the Newcastle–Ottawa Scale.

Study	Selection	Comparability	Exposure	Total score
Ucak and Unver 2020 [16]	2	2	2	6
Moyal et al. 2018 [17]	3	2	2	7
Cai et al. 2020 [22]	3	2	2	7
Çolak et al. 2021 [23]	3	2	2	7
Cai et al. 2022 [24]	3	2	2	7
Loureiro et al. 2021 [25]	3	2	2	7

## Data Availability

The data used to support the findings of this study are included within the article.
